# Sex differences in early sensorimotor processing for speech discrimination

**DOI:** 10.1038/s41598-018-36775-5

**Published:** 2019-01-23

**Authors:** David Thornton, Ashley W. Harkrider, David E. Jenson, Tim Saltuklaroglu

**Affiliations:** 1 Gallaudet University, Washington, DC 20002 USA; 2 University of Tennessee Health Science Center, Knoxville, TN, 37996 USA; 3 Elson S. Floyd College of Medicine, Washington State University, Spokane, WA 99202 USA

## Abstract

Sensorimotor activity in speech perception tasks varies as a function of context, cognitive load, and cognitive ability. This study investigated listener sex as an additional variable. Raw EEG data were collected as 21 males and 21 females discriminated /ba/ and /da/ in quiet and noisy backgrounds. Independent component analyses of data from accurately discriminated trials identified sensorimotor mu components with characteristic alpha and beta peaks from 16 members of each sex. Time-frequency decompositions showed that in quiet discrimination, females displayed stronger early mu-alpha synchronization, whereas males showed stronger mu-beta desynchronization. Findings indicate that early attentional mechanisms for speech discrimination were characterized by sensorimotor inhibition in females and predictive sensorimotor activation in males. Both sexes showed stronger early sensorimotor inhibition in noisy discrimination conditions versus in quiet, suggesting sensory gating of the noise. However, the difference in neural activation between quiet and noisy conditions was greater in males than females. Though sex differences appear unrelated to behavioral accuracy, they suggest that males and females exhibit early sensorimotor processing for speech discrimination that is fundamentally different, yet similarly adaptable to adverse conditions. Findings have implications for understanding variability in neuroimaging data and the male prevalence in various neurodevelopmental disorders with inhibitory dysfunction.

## Introduction

It is well-established that throughout the course of development and into adulthood, females generally outperform males in verbal language abilities. However, the sources of these differences are not well understood. At least in children, the development of many linguistic skills is associated with basic speech perception abilities^1,2^, yet it remains unclear if males and females exhibit basic neural differences for processing speech sounds. In addition to activating auditory regions, speech perception tasks are well-known to recruit portions of the dorsal stream (i.e. premotor/motor cortex) that may provide dynamic sensorimotor-based cognitive (e.g., attention and working memory) scaffolding for audition^3–5^. Anterior dorsal activity has shown high intra- and inter-individual variability that typically is related to cognitive demands^6^, stimulus context^6^ and cognitive abilities^7^. There are a number of reasons to suspect that a listener’s sex may be an additional source of variability. First, males have been shown to have higher grey matter density in the precentral gyrus^8^. Second, ample behavioral and neurophysiological data demonste sex differences in cognitive tasks, especially attention^9–16^. Third, behavioral^17,18^ and neuroimaging studies using various techniques^12,19–25^ have revealed sex-based differences in tasks with different attentional demands that also may apply to speech perception. Lastly, a number of communication disorders with sensorimotor deficits are more often diagnosed in males^26,27^. Despite these observations, it remains unclear if sensorimotor processing for classic speech discrimination tasks displays sexual dimorphism.

Electroencephalography (EEG) offers strong temporal resolution for precisely capturing event-related changes in neural activity across the time course of speech perception tasks. Power in both alpha (8–13 Hz) and beta (15–25 Hz) rhythms is sensitive to capturing sensorimotor function^28,29^ across the time course of a perception event, including early attentional and later working memory processes. In particular, alpha bands are well known for producing patterns of both event-related desynchronization (ERD) and synchronization (ERS); depicting evidence of both cortical activation and inhibition, respectively^30,31^. In cognitive tasks, alpha ERD commonly is linked to active stimulus processing and associated with cognitive functions including attention and working memory^32–34^. Alpha ERS is thought to reflect sensory selection^35,36^. That is, alpha ERS may demonstrate inhibition of irrelevant information during cognitive processing within a particular region of interest^31^ or an inhibitory process that allows for reallocation of resources from non-relevant to relevant cortical regions^37^. Beta (15–25 Hz) ERD most commonly is associated with sensorimotor forward modeling in execution^38,39^, observation^40^, or even imagination of movement^41^. However, beta ERD has been found in cognitive tasks that require attention or working memory^42–44^. In a number of tasks with both cognitive and motor components, time-frequency decompositions of EEG data reveal sex-based differences in alpha and beta (in addition to theta) rhythm activity recorded across frontal and central electrodes^45^.

The sensorimotor mu rhythm emanates from the anterior dorsal stream and, when identified via magnetoecncaphalography (MEG)^46^ or independent component analysis of EEG data^34,43,47,48^, is characterized by peaks in both alpha and beta frequencies^34,43,49–51^. Consistent with alpha and beta sensitivity described above, mu rhythm oscillations are responsive to cognitive processes^28,29,34,43,47,52,53^. In addition, oscillatory activity in the alpha band of the mu rhythm previously has revealed sex differences related to empathy^54^ and attentional allocation^55^. A number of studies have temporally decomposed mu rhythm oscillations to characterize sensorimotor-based cognitive activity during the time course of speech discrimination events^34,48,50^. A well-replicated finding is that following stimulus offset, both alpha and beta bands of mu rthythms desynchronize, suggesting rehearsal while stimuli are held in working memory^34,43,47,48^. Across studies, however, mu oscillatory activity prior to and during the onset of stimulus presentation (i.e., early attentional mechanisms) has not been consistent. Of interest to the current investigation, Jenson *et al*.^43^ tested predominantly female participants and found mu beta ERD alongside mu-alpha ERS during onset of the speech stimulus that became stronger in the presence of noise, suggesting inhibition of sensorimotor activity via auditory gating of noise. Using similar methods and tasks in a predominantly male control group, Saltuklaroglu *et al*.^47^ showed markedly less robust early mu-alpha ERS, suggesting inhibition of sensorimotor activity is weaker in males than females prior to the onset of the auditory speech signal. Along with previous evidence of sex differences in behavioral and neural responses during the performance of cognitive and linguistic tasks, the data from the Jenson *et al*.^43^ and Saltuklaroglu *et al*.^47^ provide some support for mu rhythm oscillations being sensitive to sexual dimorphism in speech discrimination and additional impetus for the current examination.

In the current study, it is hypothesized that sex differences will be observed in oscillations of mu rhythms in speech discrimination tasks. Based on previous findings, sexual dimorphism is most likely to be revealed in the alpha band of the mu rhythm prior to speech perception, which would highlight differences in the contributions of early inhibitory processes related to attention and auditory gating. Time frequency analyses of anterior dorsal activity during speech perception may shed further light on sources of its variability. Additionally, results will contribute to the understanding of why certain communication disorders linked to sensorimotor deficits primarily impact males.

## Methods

### Participants

42 English-speaking adults (21 females, 21 males) with a mean age of 25.73 years (range 18–43 years, SD = 5.441) were recruited from the greater Knoxville area to participate in this study. Data from all female participants and 2 of the males were collected and analyzed in Jenson *et al*.^43^. For the purposes of this study, an additional 19 age-matched males were recruited. The average ages of male and female contributors were 26.06 and 26.25 years respectively, which were not significantly different (*t* (15) = −0.613, *p* = 0.549). Participants reported no history of cognitive, communicative, or attentional disorders. Though hearing acuity was not measured, all participants indicated they could clearly hear and attend to the stimuli. The Edinburgh Handedness Inventory^56^ was used to assess handedness dominance, with all participants indicating a right-handed preference. This study was approved by the University of Tennessee Health Science Center Institutional Review Board. In accordance with the Declaration of Helsinki, all participants gave written, informed consent prior to participation.

### Stimuli

**Figure 1:**
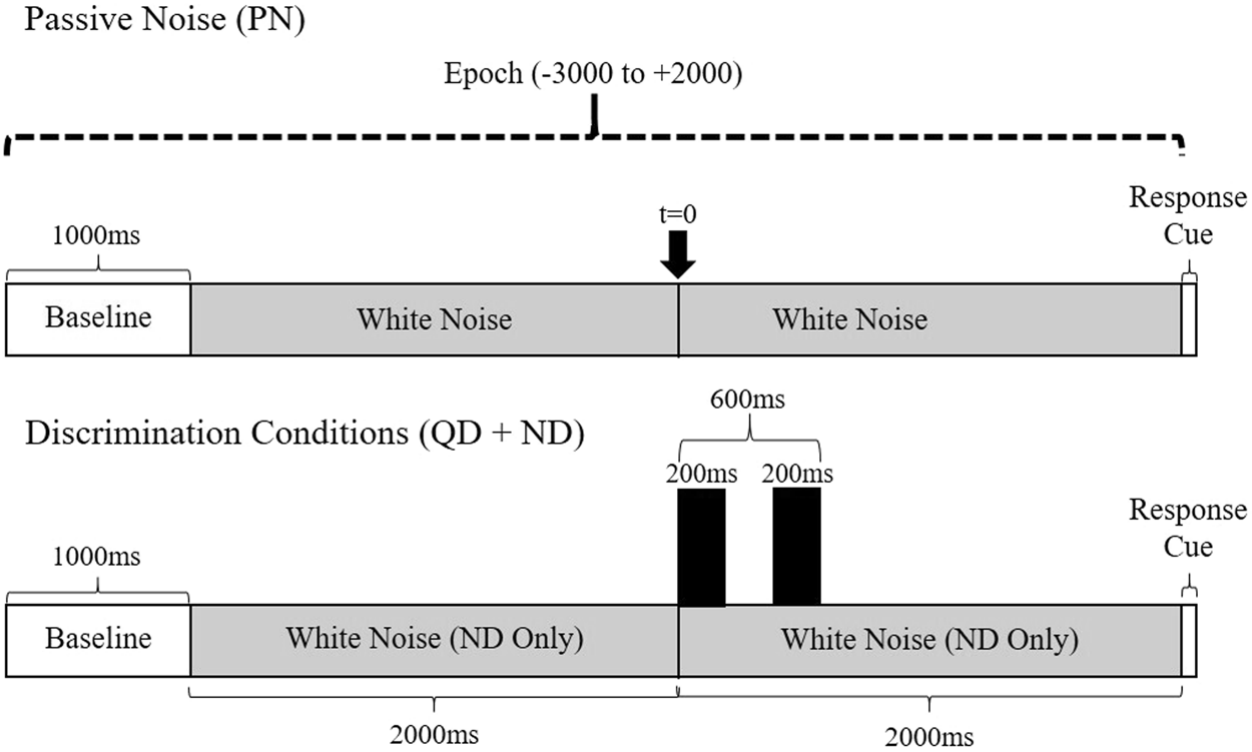
Single-trial timelines. Timelines for passive noise (PN) and the active discrimination conditions (QD and ND).

Similar to previous studies^43,48^, syllable stimuli /ba/ and /da/ were generated using AT&T naturally speaking text-to-speech software, which employs synthetic analogs of a human male speaker to create syllable utterances. To reduce potential for discrimination response bias^57^, syllable pairs were constructed so that half of the stimuli contained identical syllables (e.g. /ba//ba/) and half were different syllables (e.g. /ba/ /da/). Each syllable was 200 ms in duration, and each pair was divided by 200 ms of silence, creating a total duration of 600 ms from onset of the first syllable to offset of the second. Stimuli were low-pass filtered below 5 KHz and normalized for root-mean-square (RMS) amplitude. Syllable pairs were placed in both a quiet (quiet discrimination; QD) and noisy (noisy discrimination; ND) background for the purposes of manipulating cognitive load. For noisy discrimination, the signal-to-noise ratio (SNR) was +4 dB. A timeline depicting how syllables were presented within discrimination events is shown in Fig. 1.

### Design

Three conditions were used in a within-subject and between-subject design:

1. Control condition - Passive listening to white noise (PN).
2. Discriminate (same or different) between pairs of syllables (QD).
3. Discriminate (same or different) between pairs of syllables embedded in background noise (ND).

### Procedure

Participants sat in a reclining armchair in an electronically and magnetically shielded, double-walled, sound-treated booth for the duration of the study. Stimuli were presented at 75 dB SPL through insert earphones and responses were recorded using the Compumedics NeuroScan Stim 2 4.3.3 software system.

In the control condition, participants were asked to passively listen to white noise in each trial. This control condition has been used in previous studies and is effective for stimulating auditory attention without eliciting sensorimotor activity in neurotypical speakers^34,43,48^. In discrimination conditions, participants were asked to listen to the stimuli and indicate if the syllables in each pair were the same or different via button-press response. The cue to respond was a 100 ms, 1 KHz ‘chirp’ that occurred at the end of each trial (+2000 ms – see Fig. 1). A button-press was required in the PN as well as the discrimination conditions to help maintain attention and control for any sensorimotor activity occurring in anticipation of a motor response (i.e., button-press)^29,58^. To control for lateralization of anticipatory motor responses, participants alternated between left- and right-handed responses for the two blocks of trials presented in each condition. As such, each condition consisted of 80 trials, presented in two blocks of 40 trials, each with a left- and right-handed response. The order of conditions was randomized for each participant.

### EEG Acquisition

EEG data were collected using 68 electrode channels on an unlinked, sintered NeuroScan Quik Cap^59^. The electrode configuration was based on the extended international 10–20^60^ system, with two electromyography (EMG) and two electrocardiogram (ECG) channels. Mastoid electrodes (M1 and M2) were used as a reference for all recording electrodes. Eye movement artifact was recorded by placing electrodes on the lateral canthi of each eye (HEOG) along with the superior and inferior orbit (VEOG) of the left eye. The EMG surface electrodes were placed above and below the lips to monitor for any perilabial movement artifact.

EEG data were collected with a Synamps 2 system and Compumedics NeuroScan 4.3.3 software. Raw files were band-pass filtered (from 0.15–100 Hz) and digitized by a 24-bit analog to digital converter, sampling at 500 Hz. Continuous EEG data contained event markers representing stimulus onset and participant responses.

### EEG Data Processing

EEGLAB 2014a^61^, an open source MATLAB toolbox, was used to process continuous EEG data. The following details the steps used to process the EEG data at both the individual and group levels. Additional details regarding data processing steps and analysis methods are described below.

### Individual processing/analysis of EEG data

Data from the two 40-trial blocks for each condition underwent a series of preprocessing steps. Specifically, data were: (1) appended in order to create one, 80 trial set for each of the conditions; (2) downsampled to 256 Hz in order to reduce computational load for ICA processing; (3) filtered from 3–34 Hz to isolate mu related activity. This band pass filter was used as it captures mu frequencies of interest and eliminates other extraneous low- and high-frequency information; (4) epoched into 5000 ms segments around time point zero (stimulus onset), creating epochs that extended from −3000 to +2000 ms; (5) re-referenced to M1 and M2 (mastoid electrodes); (6) inspected for gross artifact (>200 uV), visually; and (7) cleaned of incorrect or late responses (responses entered greater than 2 s following the response cue). At least 40 epochs per participant in each condition were required in order to include the participant in the experiment. The overall average of usable trials exceeded this inclusion criterion comfortably.

### Independent component analysis (ICA)

A single set of ICA weights were obtained by concatenating the three pre-processed EEG data sets. This method ensured that an accurate comparison was made across conditions within the revealed ICs. The “extended *runica*” algorithm in EEGLAB 2014a provided the ICA training, with an initial learning rate of 0.001 with a stopping weight of 10e^−7^. Each participant returned 66 ICs following ICA decomposition, reflecting the number of recording electrodes with the mastoid reference electrodes removed (68 – 2 reference electrodes). The inverse weight matrix (W^−1^) was projected onto the spatial EEG channel configuration in order to obtain scalp maps for each IC from each participant.

### Equivalent current dipole (ECD) source localization

Equivalent current dipole (ECD) models for each component were computed following ICA decomposition. Using the brain electrical source analysis (BESA) model, dipoles were localized in the DIPFIT toolbox, a free extension of the EEGLAB toolbox^62^. Dipole processing produced a single dipole for each of the 2772 ICs (66 IC x 42 participants). Localization of produced dipoles to a cortical area requires back-projection to a signal source that likely generated the scalp potential distribution for each IC. The best forward model is computed with the goal of explaining the highest amount of scalp map variance^63^. Remaining unexplained variance is termed “residual variance” (RV) and used as a determiner of “goodness of fit” between the scalp map and projected ECD model.

### Current source density (CSD) Source Localization

Current source density (CSD) measures using standardized low-resolution brain electromagnetic tomography (sLORETA) were conducted as a reliability check for ECD sources. sLORETA relies on current source density (CSD) from recorded signals to estimate the source of activity within the brain and address the inverse problem^64^. The base head model for sLORETA is a Talairach cortical probability brain atlas that is digitized at the Montreal Neurological Institute. EEG scalp electrodes are co-registered between realistic head geometry and the BESA spherical head model^59^. This model divides the brain space into 6239 voxels, yielding a spatial resolution of 5 mm. EEG channels and the inverse weight projections were exported to sLORETA for each identified mu cluster IC. Estimates of CSD for the components within each mu cluster were revealed by mapping cross-spectra and co-registering with the coordinates from the head model. Paired *t*-tests were used to assess the statistical significance of the CSD estimates by comparing relative activity to no activation^65^. Paired (Student) *t*-tests using a smoothing parameter of 1, the common variance for all components, assessed all frequencies between 3–34 Hz. Voxels that were significant at *p* < 0.001, after establishing a corrected threshold using 5000 random permutations, were considered active across participants

### Group data analyses

Group data were analyzed using the EEGLAB STUDY module. This module is used to analyze ICA data from multiple participants across experimental conditions under different designs. STUDY allows for inclusion parameters to be implemented. Any dipole with a residual variance of 20% or below was included at this step.

### Assignment of components to mu clusters

Principal component analysis (PCA) was used to identify clusters of neural components common to all participants. Using the K-means statistical toolbox, PCA identified 35 initial clusters of EEG activity on the bases of common dipoles, spectra, and scalp maps. One left and one right mu cluster were identified. However, previous analyses^34,43,47,50^ show that PCA clustering can sometimes include components that do not fit the mu inclusion criteria. The criteria used were: (a) characteristic mu spectra, with peaks in both the alpha (8–13 Hz) and beta (15–25 Hz) frequency bands, (b) appropriate source localization to BA 2, 3, 4, 6 and (c) RV of <20%^34,43,47,48^. Components that did not fit these criteria were removed. Additionally, PCA can sometimes assign mu components that fit the criteria to neighboring (non-mu) clusters. Thus, visual inspection of the mu clusters and neighboring clusters was necessary to identify and re-assign misplaced mu components. At least two of the authors agreed on cluster membership for all components included in left and right mu clusters.

### Time-frequency analyses

Event-related spectral perturbations (ERSPs) were used to investigate changes in mu rhythm oscillatory activity. Time-frequency transforms were extracted using a Morlet sinusoidal wavelet set at 3 cycles at 3 Hz and 34 cycles at 34 Hz with a linear rise. Each trial was referenced to a 1000 ms baseline which was selected from the inter-trial interval. A surrogate distribution was constructed using 200 randomly selected latencies from within the selected inter-trial interval^61^. Changes from baseline in ERSPs were computed using a bootstrap resampling method (*p* < 0.05 uncorrected).

Within-subject and between-group differences across time and frequency were identified using permutation statistics. The significance threshold of *p* < 0.05 was implemented and, in order to control for type one error, a false discovery rate (FDR) correction was used^66^. Following an initial omnibus analysis that showed differences related to condition and group, a quiet versus noisy discrimination contrast was conducted to assess within-subject sensorimotor differences related to presence of noise.

## Results

### Discrimination Accuracy

All tasks produced over 86% accuracy in participants who contributed to mu clusters, with no significant differences in either test condition between males and females (QD: F(1, 30) = 0.153, *p* = 0.698, ND: F(1,30) = 0.038, *p* = 0.847). Only trials with correct responses within 2000 ms of stimulus offset were included in further analysis. Mean reaction times to respond are 1217 ms and 1201 ms for males for QD and ND, respectively. Mean female reaction times are 1165 ms and 1250 ms for QD and ND, respectively. These times are not statistically different (QD: F(1,30) = 0.192, *p* = 0.664, ND: F(1,30) = 1.088, *p* = 0.305). The average number of trials that were both accurately discriminated and clean, and therefore could be used in ICA were: PN = 68.26 (SD = 7.47); QD = 69.36 (SD = 7.78); ND = 66.84 (SD = 7.07).

### Mu cluster characteristics

Of the 21 females, 20 contributed to either the left or the right mu cluster. Specifically, 12 contributed to both clusters while 4 contributed only to the left and 4 only to the right. All 21 of the matched males contributed to at least one of the mu clusters. Specifically, 11 contributed to both clusters while 5 contributed only to the left and 5 only to the right.

The same proportion (16/21) of members of each sex contributed to both mu clusters. As is common with high density electrode arrays, it was possible for sensorimotor activity to be spread across multiple components, allowing participants to sometimes contribute more than one component to each cluster. The total number of mu components per cluster was 44 (22 from each sex) in the left and 60 (30 from each sex) in the right. Data from components were used for statistical spectral and time-frequency analyses in each hemisphere. Information regarding the source localization of mu clusters using the two different methods is shown in Table 1.

**Table 1:** Source information for each mu cluster.

Source Information	Left mu	Right mu
**By Group**		
Males: Mean ECD source	[−38, −10, 41] (BA 6)	[39, −9, 42] (BA 6)
Mean RV	6.94%	6.3%
Females: Mean ECD source	[−42, −7, 41] (BA 6)	[38, −8, 43] (BA 6)
Mean RV	7.53%	8.08%
Distance between sources	5 mm	1.7 mm
**Male and Females groups combined**		
Mean ECD source	[−41, −8, 42] (BA 6)	[38, −8, 42] (BA 6)
Maximum CSD source	[−40, −15, 50] (BA 4)	[40, −10, 50] (BA 6)
Distance between sources	17 mm	8.5 mm

Notes: ECD = Equivalent Current Dipole; CSD = Current Source Density; BA = Brodmann’s Area; RV = Residual Variance.

### Spectral analysis

**Figure 2:**
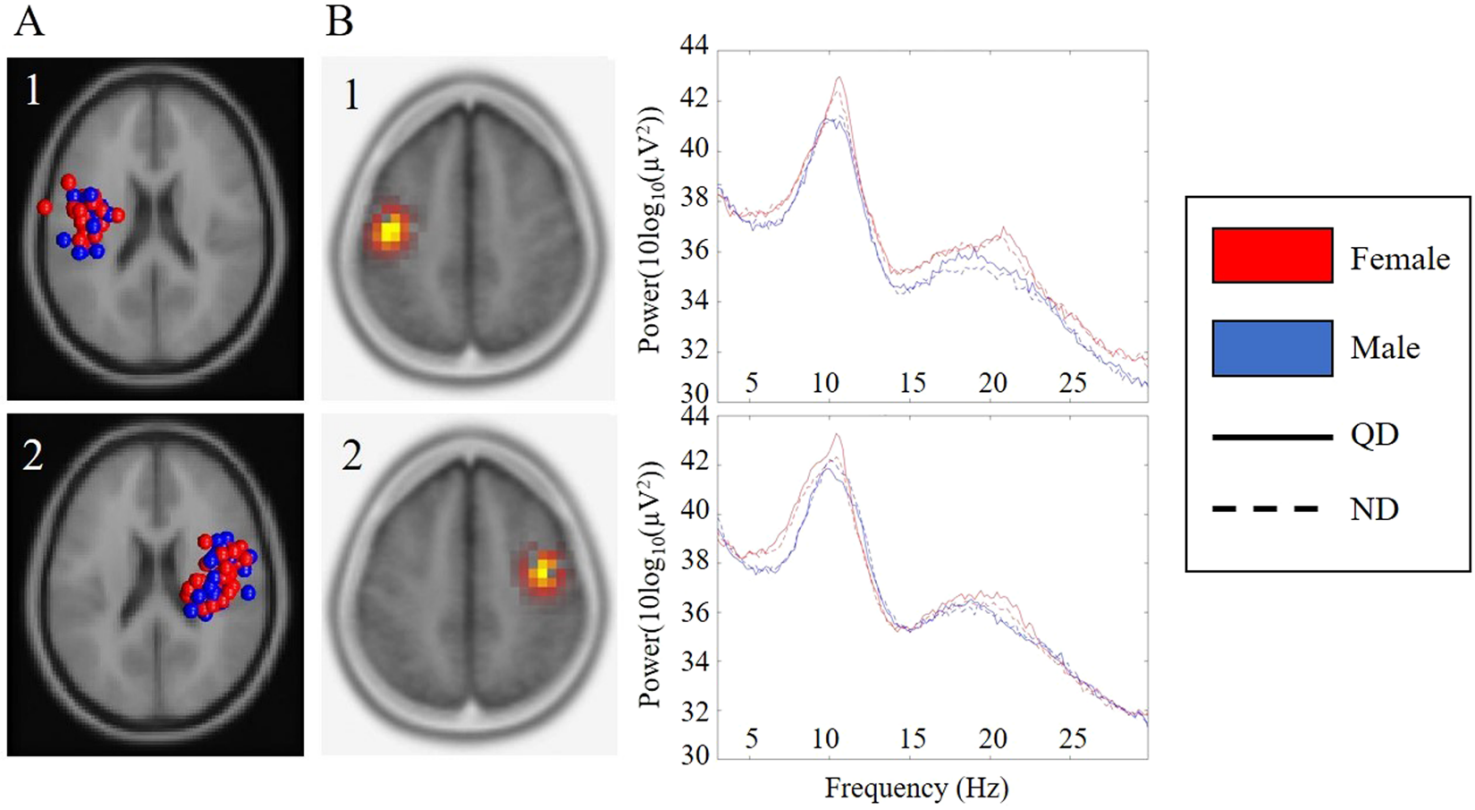
Cluster results for left and right µ components. (**A**) ECD localized dipoles for the left (1) and right (2) mu clusters (red = female, blue = male), (**B**) maximum current source density voxels (t-values), (**C**) mean spectra for left (1) and right (2) cluster components in each condition (Red = females; Blue = males; Solid line = quiet discrimination; QD); Dashed line = noisy discrimination; ND). Notes: ECD = Equivalent Current Dipole.

Average mu spectra across conditions are shown for both males and females in Fig. 2. No differences in spectral power were observed across conditions or between groups.

### Time-frequency analyses

For all conditions, patterns of mu oscillatory activity are similar in both hemispheres, suggesting bilateral sensorimotor activity. However, across conditions, patterns of significant differences in the left hemisphere are more robust.

### Within- and between-group differences

**Figure 3:**
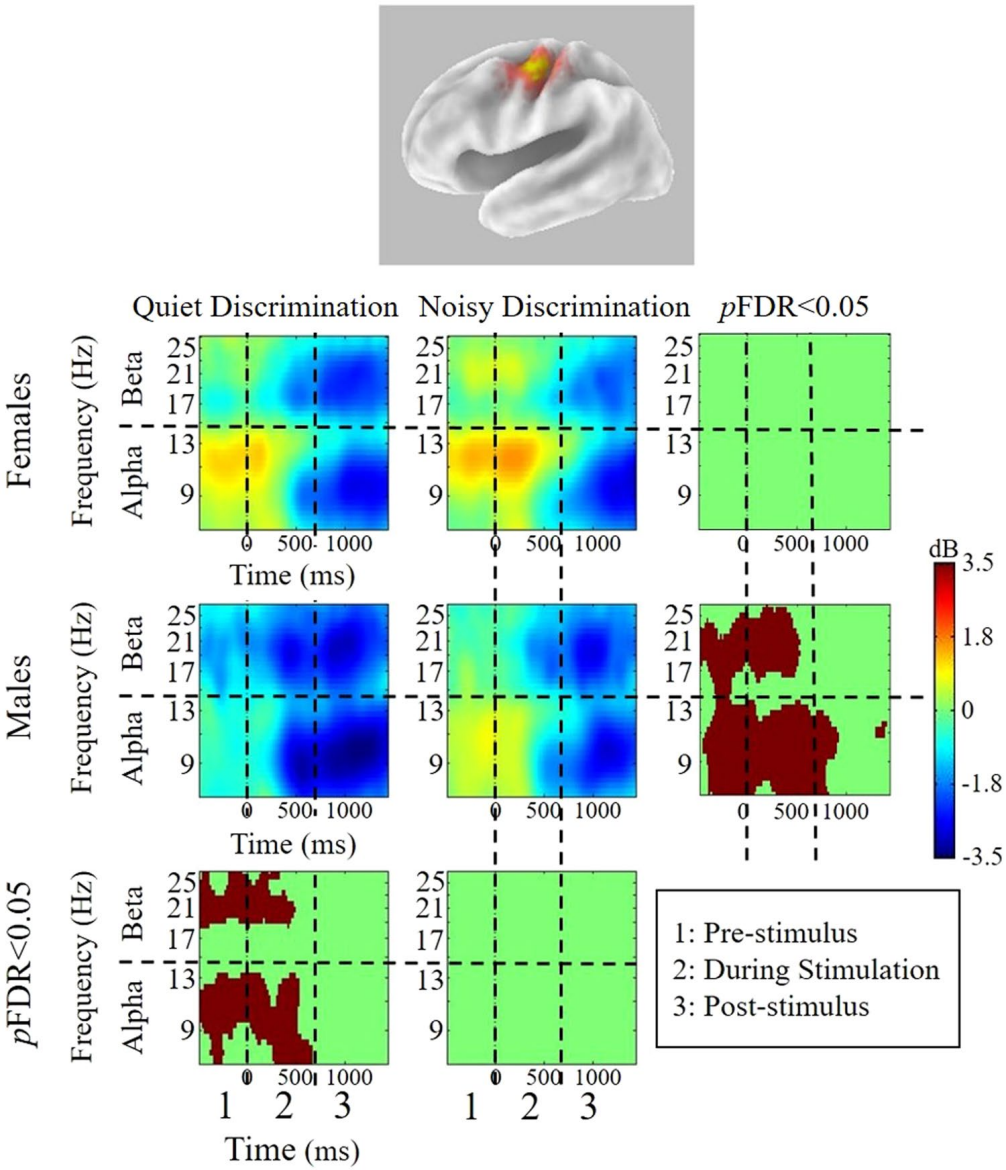
sLoreta and mean ERSPs for test conditions in the left hemisphere. sLoreta solutions for the left mu cluster, shown on a 3D Van Essen template highlighting significantly activated voxels associated by mu components, with mean ERSPs across all test conditions (warm colors indicate ERS whereas cool colors indicate ERD). For each condition, ERSPs display changes from a recorded baseline before stimulus onset (−3000 to −2000 ms before stimulus onset for each trial). The final panel in each row indicates statistical differences within each group across test conditions, while the final panel in each column indicates differences between groups within each test condition (*p*FDR < 0.05). Note: QD = Quiet Discrimination; ND = Noisy Discrimination; FDR = Corrections for False Discovery Rate; ERSP = Event-related Spectral Perturbations.

**Figure 4:**
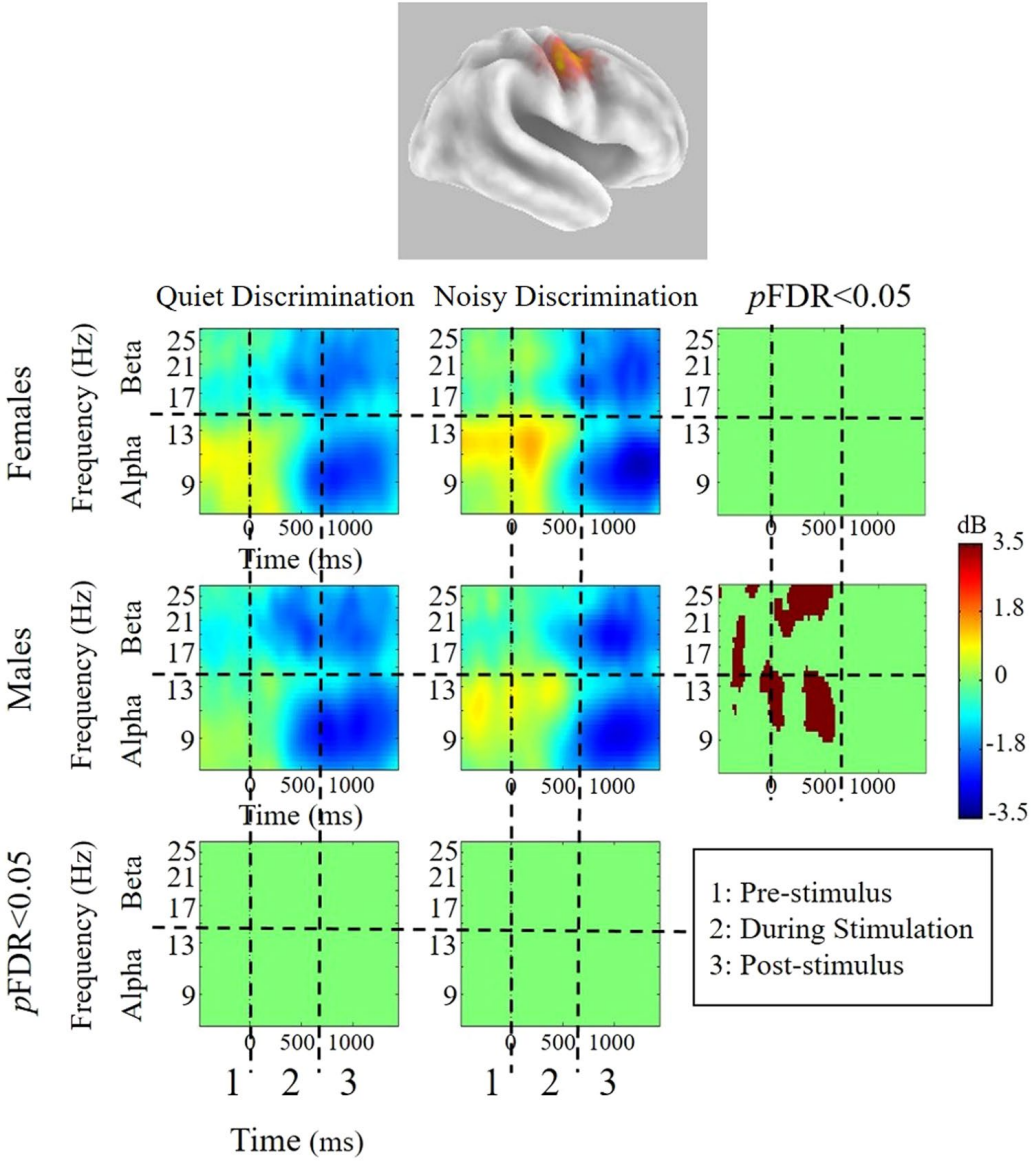
sLoreta and mean ERSPs for test conditions in the right hemisphere. sLoreta solutions for the left mu cluster, shown on a 3D Van Essen template highlighting significantly activated voxels associated by mu components, with mean ERSPs across all test conditions (warm colors indicate ERS whereas cool colors indicate ERD). For each condition, ERSPs display changes from a recorded baseline before stimulus onset (−3000 to −2000 ms before stimulus onset for each trial). The final panel in each row indicates statistical differences within each group across test conditions, while the final panel in each column indicates differences between groups within each test condition (*p*FDR < 0.05). Note: QD = Quiet Discrimination; ND = Noisy Discrimination; FDR = Corrections for False Discovery Rate; ERSP = Event-related Spectral Perturbations.

**Figure 5:**
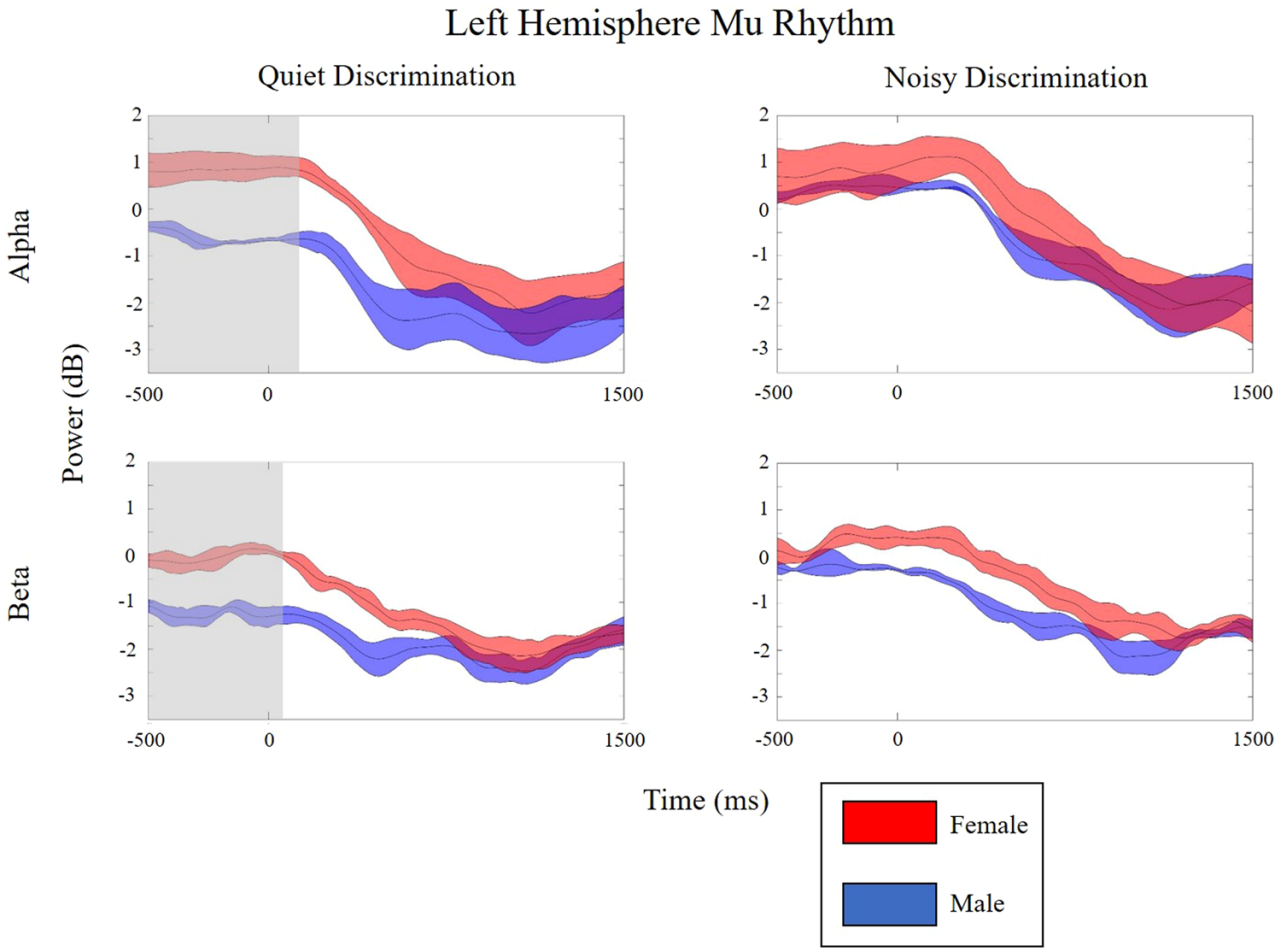
Alpha and beta contour lines showing power across time for the left hemisphere mu cluster. The middle red (female) and blue (male) lines represent mean alpha (8–13 Hz) and beta (15–25 Hz) value deviations from baseline, while shaded regions indicate the upper and lower quartile limits. Values above zero are ERS, while values below zero are ERD. Significant differences are noted by the grey regions.

**Figure 6:**
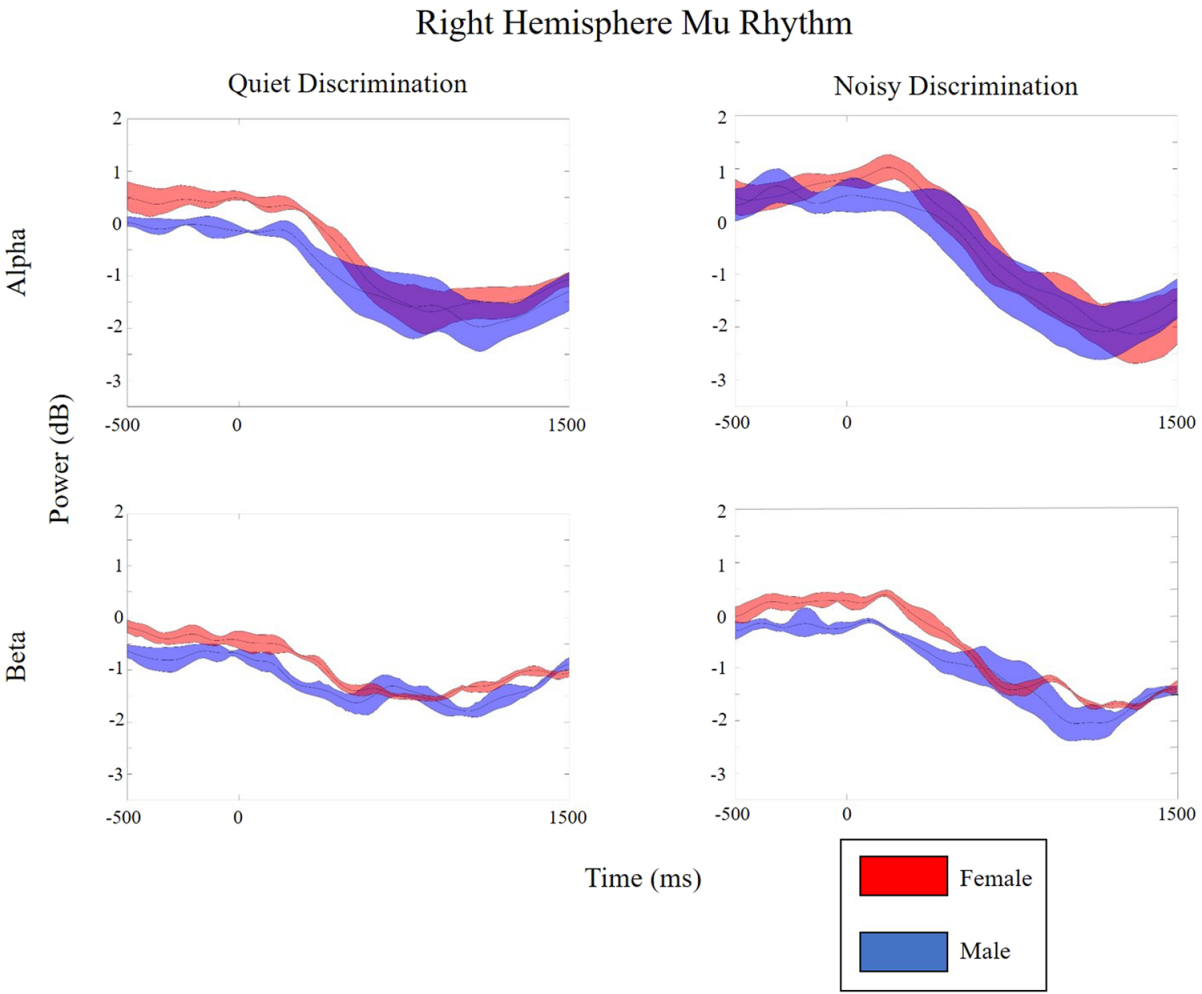
Alpha and beta contour lines showing power across time for the right hemisphere mu cluster. The middle red (female) and blue (male) lines represent mean alpha (8–13 Hz) and beta (15–25 Hz) value deviations from baseline, while shaded regions indicate the upper and lower quartile limits. Values above zero are ERS, while values below zero are ERD. Significant differences are noted by grey regions.

Within-subject differences (*p*FDR < 0.05) were found before, during, and after stimulus presentation for both sexes when compared to the control condition (PN). Figures 3 and 4 show time-frequency decompositions of left and right mu rhythm oscillatory activity across test conditions and between groups. In the left hemisphere, within-subject differences were only observed in males. Between-group differences were found only in the QD condition, where females show stronger early mu-alpha ERS and males show stronger mu-beta ERD. In the right hemisphere, patterns of ERSP activity were similar to those found in the left hemisphere. Within-subject differences were again only found in males, though the difference was less robust than in the left hemisphere (i.e., fewer significant time-frequency voxels). No between-group differences were observed in the right hemisphere. Figures 5 and 6 display directional differences in mu-alpha and mu-beta spectral power across the time-course of the stimulus in each test condition for the left and right mu clusters, respectively.

## Discussion

The goal of this study was to investigate sexual dimorphism in sensorimotor-based cognitive activity in speech discrimination. Matched male and female cohorts demonstrate similar high levels of discrimination accuracy. In addition, they contribute similar numbers of components to left and right mu clusters with similar spectral amplitudes and cortical source localization (BA 6/4). Current mu rhythm sources are consistent with those previously identified using ICA^43,48,51^, known generators of mu rhythms^28,29^ and with regions of the anterior dorsal stream shown to activate during speech perception^67–69^. Basic groups similarities in accuracy and mu spectral amplitudes allow for time-frequency decompositons to test the research hypothesis that, across the time course of the stimulus presentation, males and females employ different cognitive-based sensorimotor strategies in speech discrimination tasks.

For both groups, the two speech discrimination conditions yield bilateral oscillatory changes compared to the control condition, indicating task-related sensorimotor activity prior to, during, and following syllable perception. Overall, mu activity in the left (Figs 3 and 5) and right (Figs 4 and 6) hemisphere are characterized by similar oscillatory patterns, though weaker in the right hemisphere. This is consistent with findings showing that sensorimotor transformations for speech occur bilaterally^70^ but are left-hemisphere dominant^5,71^. Within-subject differences related to experimental condition are found in both left and right mu rhythm activity (though more significant voxels were found in the left). However, between-group differences and within-subject differences between the two experimental conditions (QD and ND) are only found in the left hemisphere. As such, left hemisphere findings are the current focus.

### Is sensorimotor activity during speech perception/discrimination sexually dimorophic?

Group differences occur in the quiet discrimination (QD) condition. Prior to and during syllable presentation, females display stronger mu-alpha ERS than males, whereas males display stronger mu-beta ERD than females (Figs 3 and 5). Hence, the two sexes appear to employ different early attentional strategies when accurately discriminating in quiet (i.e., optimal listening) backgrounds. According to models of speech perception such as analysis-by-synthesis, early attentional mechanisms provide top-down predictive codes in the form of sensorimotor forward models that help constrain the forthcoming sensory analysis^72–75^. As beta ERD is considered an index of forward modeling, current data suggest that males are more prone to use this early sensorimotor strategy when repeatedly discriminating. Similar early predictive mu-beta ERD has been observed in other speech perception studies^43,48^ and during auditory beat tracking^76,77^ or tracking task relevant information^78^. In contrast to early mu-beta ERD observed in males, females exhibit reduced mu-beta ERD alongside robust mu-alpha ERS. Thus, in lieu of using motor-based projections to predict forthcoming syllables, females appear to inhibit early sensorimotor activity. In cognitive tasks, alpha ERS conveying cortical inhibition is often associated with gating of task irrelevant information^79,80^ or the re-allocation of cognitive resources to cortical regions involved in processing^37^. Though it may not be possible to completely disentangle these two functions, in quiet listening conditions (without noise to gate) the latter explanation seems more plausible. This inhibitory activity in anterior dorsal regions perhaps allows cortical resources to be reallocated to regions involved in sensory analysis (e.g., auditory regions). Inhibition of sensorimotor cortex related to cognitive resource reallocation has been observed in working memory load^81,82^ and attention-based tasks^31,36,79,83^. Together, data clearly highlight sexual dimorphism in early sensorimotor-based cognitive strategies for repeated speech discrimination in optimal conditons.

In the noisy background (ND) versus in quiet, males and females both exhibit increases in early mu-alpha oscillatory activity when discriminating speech. For males, this pattern constitutes a significant shift in sensorimotor processing strategy from predictive (mu-beta ERD) in the quiet background to inhibitory (mu-alpha ERS) in the noisy background (Figs 3 and 5). In contrast, females appear to use the same sensorimotor processing strategy in noise as they do in quiet, showing slightly stronger early inhibitory activity during discrimination in noise, though the difference does not reach significance (Figs 3 and 5). Thus, in the presence of noise, a general increase in mu-alpha ERS may be related to inhibitory gating of the noise^84^. Sensory gating of irrelevant stimuli is thought to aid performance and often observed with cognitive load increases^35^. The fact that it occurs early is consistent with the inhibitory effect helping to provide top-down active sensing, as described when overcoming the ‘cocktail party problem’^85^. Saltuklaroglu *et al*. (2017)^47^ recently reported a similar shift in early mu oscillatory activity in speech discrimination when (predominantly) males switched from quiet to noisy listening. Interestingly, a matched group of (predominantly) males who stutter did not demonstrate this shift, failing to show inhibitory activity in the presence of noise. This finding was linked to auditory gating^86^ and sensorimotor adaptation deficits that are known to characterize stuttering^87,88^.

Though findings demonstrate generally stronger early inhibitory activity (mu-alpha ERS) in females and stronger early predictions (mu-beta ERD) in males, the absence of sexual dimorphism in discrimination accuracy suggests that neither of the early sensorimotor processing strategies offer a functional sex-related advantage. Both groups display similar levels of strong mu-alpha and mu-beta ERD after stimulus offset, activity often attributed to later processing (e.g., covert replay)^43,89,90^. It is possible that this late activity provides the functional support for accurate speech discrimination although additional comparisons of data from correct versus incorrect trials are necessary to verify. Considering ongoing debates^6,75,91^ regarding the functional role of sensorimotor processing in speech perception, it also is necessary to consider that all sensorimotor activity is epiphenomenal and that auditory discrimination is accomplished through sensory analysis in temporal regions. Regardless, of functionality, the finding of sexual dimorphism in sensoriomotor processing is consistent with a recent systematic review of sex differences in child language development, indicating sexual dimorphism in linguistic strategies that do not produce a functional advantage^92^. Thus, it is not clear how these findings are related to language abilities in neurotypical populations, though there may be implications for those with neurophysiological disorders.

### Sensorimotor inhibitory activity links to developmental neurophysiological disorders

Mu rhythm oscillatory activity also is under the influence of the basal ganglia as the sensorimotor regions from which they emanate are sites of integration for two main basal ganglia based loops^93–95^. Basal ganglia help provide inhibitory function critical to selection and processes that modulate and stabilize sensory experience and fluid movements^31,37,96^. Bonstrup *et al*.^97^ revealed the efficacy of measuring alpha ERS from motor regions (similar to our mu-alpha) to demonstrate reduced inhibitory function found with an age-related decline in basal ganglia function, suggesting that inhibitory power decreases with age and leads to reduced inhibitory mechanisms^97^. Interestingly, in addition to stuttering^47,98^, inhibitory deficits also are linked to a number of neurodevelopmental disorders, such as autism^99^, Tourette’s syndrome^100–102^, and attention deficit/hyperactivity disorders^103^. All are documented to have a higher prevalence in males than females^104^. Therefore, sex-based differences in inhibitory activity examined in simple cognitive tasks, such as those herein, may in some way be related to the male prevalence of developmental neurophysiological disorders. If so, measurement of mu-alpha power in tasks known to recruit inhibitory processes (e.g., speech discrimination in noise) may prove useful for identifying or measuring severity of inhibitory dysfunction. However, to accomplish this, appropriate control data separated by sex are critical, as grouped control data that include both males and females may be confounded by sex differences.

### Relevance to neuroimaging findings in speech perception

The current findings may be indicative of an additional source of variability in the activity of the anterior dorsal stream during speech perception. Previously uncovered sources of variability (e.g. context, individual cognitive ability, and task difficulty) mainly have been identified using fMRI^7,105,106^; an imaging technique with excellent spatial resolution. The detection of sexual dimorphism in sensorimotor processing was made possible by the superior temporal resolution of EEG. In contrast to fMRI, this EEG technique allows the cognitive contributions to sensorimotor activity surrounding speech perception to be delineated clearly in syllable discrimination. It will be of further interest to examine whether similar sexual dimorphism exists in passive speech perception tasks where cognitive demands are drastically reduced. Passive speech perception tasks have produced a number of conflicting results with regards to the presence or absence of sensorimotor activity, spurring ongoing debates regarding the nature and functional role of sensorimotor activity in speech perception^107^. The current data suggest that sex of the participant may be an additional variable to consider in such debates.

It remains somewhat unclear how the current findings compare to those from fMRI. Several studies have noted that increased BOLD responses seems to be correlated negatively with alpha power^108,109^, which seems logical as both alpha ERD and fMRI BOLD signals are indicators of cortical activity. There also is evidence to suggest a similar relationship in the beta band^110^. As such, fMRI findings of increased sensorimotor activity in discrimination tasks versus passive listening might be due to the strong late activity when stimuli are held in working memory; the time period that corresponds with the current findings of strong mu-alpha and mu-beta ERD. The relationship between mu-alpha and BOLD activity also has been found when mu-alpha becomes synchronized. That is, spikes in mu-alpha power suggesting cortical inhibition have been found in the presence of a negative BOLD signal^111,112^. However, it is unclear if the negative BOLD signal represents a true inhibitory response^112,113^. As such it remains an open question as to whether observing mu-alpha ERS across the time course of an EEG event would be associated with reduced overall BOLD activity or, as some have suggested, have an additive effect^114^.

### Limitations

Though 41/42 participants contributed at least one mu component in the left or right hemisphere, bilateral components were not identified in many participants. The main reason is the strict inclusion criteria (correct localization, low RV, and characteristic spectra) used in this and previous studies^34,43^ to ensure the veracity of all mu components. Thus, though nearly all particpants produced bilateral ‘mu-like’ components, some were not included because they did not meet the criteria. The main reasons for this are (1) signal noise, that can be an issue even after ICA is applied, and compromise the spectal integrity of the mu rhythm and (2) failure of the mu-like rhythms to localize to sensorimotor regions (i.e., known mu generators). That said, data from the participants included in each cluster were sufficient to reveal within- and between-group differences, even with the conservative criteria for data inclusion. Lastly, this study only examined independent activity from left and right anterior aspects of the dorsal stream. The left hemisphere appeared to be more involved in syllable discrimination as indicated by increased oscillatory power in time-frequency analysis and significant findings in the left hemisphere only. However, statistical comparisions were not made of activity between conditions, mainly due to the relative few participants that contributed to both left and right mu clusters. Future studies that seek to make such comparisons and extract other (e.g., auditory components) to measure functional connectivity should apply individualized head models that enable more accurate source localization

## Conclusions

By identifying EEG mu components using ICA and adding a temporal dimension in measurement, sex-based differences in sensorimotor activity during speech discrimination are revealed. Decomposition of the mu rhythm over time makes it possible to obtain rich measures of sensorimotor processing that consider how oscillations in alpha and beta frequencies align and dissociate across an event^115^. Here, we show that males and females display differences in early sensorimotor activation during speech discrimination in quiet backgrounds, suggesting the use of different attentional mechanisms in optimal conditions. The early inhibitory versus predictive patterns in females and males during optimal listening conditions, respectively, may have implications for understanding (1) previously described sexual dimorphism in various verbal cognitive functions and (2) the male prevalence of neurodevelopmental disorders with inhibitory dysfunction. In noisy conditions, data reveal evidence of sensory gating in both sexes. Together, findings suggest that males and females exhibit early sensorimotor processing for speech discrimination that is fundamentally different in quiet, yet similarly adaptable to adverse conditions. Further, sex differences appear unrelated to discrimination accuracy, suggesting neither early sensorimotor-based cognitive strategy provides a sex-related behavioral advantage. Finally, the nature of the mu rhythm and the precise temporal resolution afforded by this EEG technique provides an excellent means of mapping neural activity to behavior in light of cognitive requirements.

## Data Availability

The datasets generated during and/or analysed during the current study are available from the corresponding author on reasonable request.

## References

[1] Ziegler JC, Pech-Georgel C, George F, Alario F-X, Lorenzi C (2005). Deficits in speech perception predict language learning impairment. Proc. Natl. Acad. Sci..

[2] Kuhl PK, Conboy BT, Padden D, Nelson T, Pruitt J (2005). Early speech perception and later language development: Implications for the ‘critical period’. Lang. Learn. Dev..

[3] Rauschecker JP (2012). Ventral and dorsal streams in the evolution of speech and language. Front. Evol. Neurosci..

[4] Saur D (2008). Ventral and dorsal pathways for language. Proc. Natl. Acad. Sci..

[5] Hickok G, Poeppel D (2004). Dorsal and ventral streams: A framework for understanding aspects of the functional anatomy of language. Cognition.

[6] Pulvermüller F (2013). How neurons make meaning: Brain mechanisms for embodied and abstract-symbolic semantics. Trends Cogn. Sci..

[7] Szenkovits G, Peelle JE, Norris D, Davis MH (2012). Individual differences in premotor and motor recruitment during speech perception. Neuropsychologia.

[8] Ruigrok ANV (2013). A meta-analysis of sex differences in human brain structure. Neurosci. Biobehav. Rev..

[9] Bosco A, Longoni AM, Vecchi T (2004). Gender effects in spatial orientation: Cognitive profiles and mental strategies. Appl. Cogn. Psychol..

[10] Duff SJ, Hampson E (2001). A sex difference on a novel spatial working memory task in humans. Brain Cogn..

[11] Francisco, S., Diego, S., Corporation, T. P. & Antonio, S. Sex differences in verbal learning. *J. Clin. Psychol*. **1** (1986).

[12] Gur RC (2000). An fMRI study of sex differences in regional activation to a verbal and a spatial task. Brain Lang..

[13] Gur RC (1999). Sex differences in brain gray and white matter in healthy young adults: correlations with cognitive performance. J. Neurosci..

[14] Hyde JS (2016). Sex and cognition: Gender and cognitive functions. Curr. Opin. Neurobiol..

[15] Ingalhalikar M (2014). Sex differences in the structural connectome of the human brain. Proc. Natl. Acad. Sci. USA.

[16] Li R (2014). Why women see differently from the way men see? A review of sex differences in cognition and sports. J. Sport Heal. Sci..

[17] Hansen S (2011). Inhibitory control and empathy-related personality traits: Sex-linked associations. Brain Cogn..

[18] Evans KL, Hampson E (2015). Sex differences on prefrontally-dependent cognitive tasks. Brain Cogn..

[19] Weiss EM, Kemmler G, Deisenhammer EA, Fleischhacker WW, Delazer M (2003). Sex differences in cognitive functions. Pers. Individ. Dif..

[20] Steffensen SC (2008). Gender-selective effects of the P300 and N400 components of the visual evoked potential. Vision Res..

[21] Yuan J, He Y, Qinglin Z, Chen A, Li H (2008). Gender differences in behavioral inhibitory control: ERP evidence from a two-choice oddball task. Psychophysiology.

[22] Neuhaus AH (2009). Spatiotemporal mapping of sex differences during attentional processing. Hum. Brain Mapp..

[23] Kuptsova SV, Ivanova MV, Petrushevsky AG, Fedina ON, Zhavoronkova LA (2015). Sex-related differences in task switching: An fMRI study. Hum. Physiol..

[24] Pavlova MA, Sokolov AN, Bidet-Ildei C (2015). Sex differences in the neuromagnetic cortical response to biological motion. Cereb. Cortex.

[25] Ramos-Loyo J, Angulo-Chavira A, Llamas-Alonso LA, González-Garrido AA (2016). Sex differences in emotional contexts modulation on response inhibition. Neuropsychologia.

[26] Baron-Cohen, S. *et al*. Why are Autism Spectrum conditions more prevalent in Males? *PLoS Biol*. **9** (2011). 10.1371/journal.pbio.1001081 PMC3114757 21695109

[27] Montag C (2015). Prenatal testosterone and stuttering. Early Hum. Dev..

[28] Schnitzler A, Gross J, Timmermann L (2000). Synchronised oscillations of the human sensorimotor cortex. Acta Neurobiol. Exp. (Wars)..

[29] Hari R (2006). Action-perception connection and the cortical mu rhythm. Prog. Brain Res..

[30] Pfurtscheller, G. & Lopes, F. H. Event-related EEG/MEG synchronization and desynchronization: basic principles. **110**, 1842–1857 (1999). 10.1016/s1388-2457(99)00141-8 10576479

[31] Klimesch W, Sauseng P, Hanslmayr S (2007). EEG alpha oscillations: The inhibition-timing hypothesis. Brain Res. Rev..

[32] Weisz N, Hartmann T, Müller N, Lorenz I, Obleser J (2011). Alpha rhythms in audition: Cognitive and clinical perspectives. Front. Psychol..

[33] Zumer, J. M., Scheeringa, R., Schoffelen, J. M., Norris, D. G. & Jensen, O. Occipital alpha activity during stimulus processing gates the information flow to object-selective cortex. *PLoS Biol*. **12** (2014). 10.1371/journal.pbio.1001965 PMC4205112 25333286

[34] Thornton, D., Harkrider, A. W., Jenson, D. & Saltuklaroglu, T. Sensorimotor activity measured via oscillations of EEG mu rhythms in speech and non-speech discrimination tasks with and without segmentation demands. *Brain Lang*., 10.1016/j.bandl.2017.03.011 (2017). 10.1016/j.bandl.2017.03.011 28431691

[35] Foxe JJ, Snyder AC (2011). The role of alpha-band brain oscillations as a sensory suppression mechanism during selective attention. Front. Psychol..

[36] Haegens S, Luther L, Jensen O (2012). Somatosensory Anticipatory Alpha Activity Increases to Suppress Distracting Input. J. Cogn. Neurosci..

[37] Jensen O, Mazaheri A (2010). Shaping functional architecture by oscillatory alpha activity: gating by inhibition. Front. Hum. Neurosci..

[38] Elisabeth Kilavik, B., Zaepffel, M., Brovelli, A., MacKay, W. A. & Riehle, A. The ups and downs of beta oscillations in sensorimotor cortex., 10.1016/j.expneurol.2012.09.014 (2012). 10.1016/j.expneurol.2012.09.014 23022918

[39] Quandt LC, Marshall PJ, Shipley TF, Beilock SL, Goldin-Meadow S (2012). Sensitivity of alpha and beta oscillations to sensorimotor characteristics of action: An EEG study of action production and gesture observation. Neuropsychologia.

[40] Berends HI, Wolkorte R, Ijzerman MJ, Van Putten MJAM (2013). Differential cortical activation during observation and observation-and-imagination. Exp. Brain Res..

[41] de Lange F, Jensen O, Bauer M, Toni I (2008). Interactions between posterior gamma and frontal alpha/beta oscillations during imagined actions. Front. Hum. Neurosci..

[42] Gola M, Magnuski M, Szumska I, Wróbel A (2013). EEG beta band activity is related to attention and attentional deficits in the visual performance of elderly subjects. Int. J. Psychophysiol..

[43] Jenson D (2014). Temporal dynamics of sensorimotor integration in speech perception and production: Independent component analysis of EEGdata. Front. Psychol..

[44] Lundqvist M (2015). Gamma and Beta Bursts Underlie Working Memory. Neuron.

[45] Georgiev S, Minchev Z, Christova C, Philipova D (2011). Gender event-related brain oscillatory differences in normal elderly population EEG. Int. J. Bioautomation.

[46] Cheyne DO (2013). MEG studies of sensorimotor rhythms: A review. Exp. Neurol..

[47] Saltuklaroglu, T., Harkrider, A. W., Thornton, D., Jenson, D. & Kittilstved, T. EEG Mu (µ) rhythm spectra and oscillatory activity differentiate stuttering from non-stuttering adults. *Neuroimage*10.1016/j.neuroimage.2017.04.022 (2017). 10.1016/j.neuroimage.2017.04.022 PMC5569894 28400266

[48] Bowers, A., Saltuklaroglu, T., Harkrider, A. & Cuellar, M. Suppression of the µ rhythm during speech and non-speech discrimination revealed by independent component analysis: Implications for sensorimotor integration in speech processing. *PLoS One* **8** (2013). 10.1371/journal.pone.0072024 PMC3750026 23991030

[49] Pineda JA (2013). EEG sensorimotor correlates of translating sounds into actions. Front Neurosci..

[50] Jenson D, Harkrider AW, Thornton D, Bowers AL, Saltuklaroglu T (2015). Auditory cortical deactivation during speech production and following speech perception: an EEG investigation of the temporal dynamics of the auditory alpha rhythm. Front. Hum. Neurosci..

[51] Cuellar M (2016). Time–frequency analysis of the EEG mu rhythm as a measure of sensorimotor integration in the later stages of swallowing. Clin. Neurophysiol..

[52] Pineda, J. The functional significance of mu rhythms: translating “seeing” and “hearing” into “doing”. *Brain Res. Rev* (2005). 10.1016/j.brainresrev.2005.04.005 15925412

[53] Fox NA (2016). Assessing human mirror activity with EEG mu rhythm: A meta-analysis. Psychol. Bull..

[54] Cheng Y (2008). Gender differences in the mu rhythm of the human mirror-neuron system. PLoS One.

[55] Popovich C, Dockstader C, Cheyne D, Tannock R (2010). Sex differences in sensorimotor mu rhythms during selective attentional processing. Neuropsychologia.

[56] Oldfield RC (1971). The assessment and analysis of handedness: The Edinburgh Inventory. Neuropsychologia.

[57] Venezia JH, Saberi K, Chubb C, Hickok G (2012). Response bias modulates the speech motor system during syllable discrimination. Front. Psychol..

[58] Graimann B, Pfurtscheller G (2006). Quantification and visualization of event-related changes in oscillatory brain activity in the time-frequency domain. Prog. Brain Res..

[59] Towle VL (1993). The spatial location of EEG electrodes - Locating the best-fitting sphere relative to cortical anatomy. Electroencephalogr. Clin. Neurophysiol..

[60] Jasper HH (1958). The ten-twenty electrode system of the International Federation. Electroencephalogr. Clin. Neurophysiol..

[61] Makeig S, Debener S, Onton J, Delorme A (2004). Mining event-related brain dynamics. Trends Cogn. Sci..

[62] Oostenveld R, Oostendorp TF (2002). Validating the boundary element method for forward and inverse EEG computations in the presence of a hole in the skull. Hum. Brain Mapp..

[63] Delorme, A., Palmer, J., Onton, J., Oostenveld, R. & Makeig, S. Independent EEG sources are dipolar. *PLoS One* **7** (2012). 10.1371/journal.pone.0030135 PMC3280242 22355308

[64] Pascual-Marqui, R. D. Standardized low resolution brain electromagnetic tomography (sLORETA): technical details. *Methods Find. Exp. Clin. Pharmacol*. 1–16 841 [pii] (2002). 12575463

[65] Grin-Yatsenko VA, Baas I, Ponomarev VA, Kropotov JD (2010). Independent component approach to the analysis of EEG recordings at early stages of depressive disorders. Clin. Neurophysiol..

[66] Benjamini Y, Hochberg Y (2000). On the adaptive control of the false discovery fate in multiple testing with independent statistics. J. Educ. Behav. Stat..

[67] Pekkola J (2006). Perception of matching and conflicting audiovisual speech in dyslexic and fluent readers: An fMRI study at 3 T. Neuroimage.

[68] Ojanen V (2005). Processing of audiovisual speech in Broca’s area. Neuroimage.

[69] Specht K (2014). Neuronal basis of speech comprehension. Hear. Res..

[70] Cogan GB (2014). Sensory-motor transformations for speech occur bilaterally. Nature.

[71] Hickok G, Poeppel D (2000). Towards a functional neuroanatomy of speech perception. Trends Cogn. Sci..

[72] Skipper JI, Van Wassenhove V, Nusbaum HC, Small SL (2007). Hearing lips and seeing voices: How cortical areas supporting speech production mediate audiovisual speech perception. Cereb. Cortex.

[73] Skipper JI, Nusbaum HC, Small SL (2005). Listening to talking faces: Motor cortical activation during speech perception. Neuroimage.

[74] Callan D, Callan A, Gamez M, Maki T, Kawato M (2010). Premotor cortex mediates perceptual performance. Neuroimage.

[75] Skipper JI, Devlin JT, Lametti DR (2017). The hearing ear is always found close to the speaking tongue: Review of the role of the motor system in speech perception. Brain Lang..

[76] Fujioka T, Ross B, Trainor LJ (2015). Beta-band oscillations represent auditory beat and its metrical hierarchy in perception and imagery. J. Neurosci..

[77] Arnal LH (2012). Cortical oscillations and sensory predictions. Trends Cogn. Sci..

[78] Saleh M, Reimer J, Penn R, Ojakangas C, Hatsopoulos N (2011). Fast and slow oscillations in human primary motor cortex predict oncoming behaviorally relevant cues. Neuron.

[79] Klimesch W (2012). Alpha-band oscillations, attention, and controlled access to stored information. Trends Cogn. Sci..

[80] Hanslmayr S, Gross J, Klimesch W, Shapiro KL (2011). The role of alpha oscillations in temporal attention. Brain Res. Rev..

[81] Chen Y, Huang X (2016). Modulation of alpha and beta oscillations during an n-back task with varying temporal memory load. Front. Psychol..

[82] Bonnefond M, Jensen O (2012). Alpha oscillations serve to protect working memory maintenance against anticipated distracters. Curr. Biol..

[83] Haegens S, Handel BF, Jensen O (2011). Top-down controlled alpha band activity in somatosensory areas determines behavioral performance in a discrimination task. J. Neurosci..

[84] Strauss A, Woestmann M, Obleser J (2014). Cortical alpha oscillations as a tool for auditory selective inhibition. Front. Hum. Neurosci..

[85] Schroeder CE, Wilson DA, Radman T, Scharfman H, Lakatos P (2010). Dynamics of Active Sensing and perceptual selection. Curr. Opin. Neurobiol..

[86] Kikuchi Y (2011). Spatiotemporal signatures of an abnormal auditory system in stuttering. Neuroimage.

[87] Sengupta R, Shah S, Gore K, Loucks T, Nasir SM (2016). Anomaly in neural phase coherence accompanies reduced sensorimotor integration in adults who stutter. Neuropsychologia.

[88] Daliri, A., Wieland, E. A., Cai, S., Guenther, F. H. & Chang, S.-E. Auditory-motor adaptation is reduced in adults who stutter but not in children who stutter. *Dev. Sci*. e12521 10.1111/desc.12521 (2017). 10.1111/desc.12521 PMC5581739 28256029

[89] Burton MW, Small SL (2006). Functional neuroanatomy of segmenting speech and nonspeech. Cortex.

[90] LoCasto PC, Krebs-Noble D, Gullapalli RP, Burton MW (2004). An fMRI investigation of speech and tone segmentation. J. Cogn. Neurosci..

[91] Hickok G, Poeppel D (2007). The cortical organization of speech processing. Nat. Rev. Neurosci..

[92] Etchell A (2018). A systematic literature review of sex differences in childhood language and brain development. Neuropsychologia.

[93] Band GP, van Boxtel GJ (1999). Inhibitory motor control in stop paradigms: review and reinterpretation of neural mechanisms. Acta Psychol. (Amst)..

[94] Dillon DG, Pizzagalli DA (2007). Inhibition of action, thought, and emotion: A selective neurobiological review. Appl. Prev. Psychol. J. Am. Assoc. Appl. Prev. Psychol..

[95] Leisman G, Braun-Benjamin O, Melillo R (2014). Cognitive-motor interactions of the basal ganglia in development. Front. Syst. Neurosci..

[96] Okun M, Lampl I (2008). Instantaneous correlation of excitation and inhibition during ongoing and sensory-evoked activities. Nat. Neurosci..

[97] Bonstrup M, Hagemann J, Gerloff C, Sauseng P, Hummel FC (2015). Alpha oscillatory correlates of motor inhibition in the aged brain. Front. Aging Neurosci..

[98] Daliri A, Max L (2015). Electrophysiological evidence for a general auditory prediction deficit in adults who stutter. Brain Lang..

[99] Murphy JW, Foxe JJ, Peters JB, Molholm S (2014). Susceptibility to distraction in autism spectrum disorder: Probing the integrity of oscillatory Alpha-Band Suppression Mechanisms. Autism Res..

[100] Comings DE, Comings BG (1985). Tourette syndrome: clinical and psychological aspects of 250 cases. Am. J. Hum. Genet..

[101] Ziemann, U., Paulus, W. & Rothenberger, A. Decreased motor inhibition in Tourette’ s disorder: Evidence from transcranial magnetic stimulation. *Am.J Psychiatry* 1277–1284 (1997). 10.1176/ajp.154.9.1277 9286189

[102] Yang J (2016). The prevalence of diagnosed tourette syndrome in Canada: A national population-based study. Mov. Disord..

[103] Vollebregt MA, Zumer JM, Ter Huurne N, Buitelaar JK, Jensen O (2016). Posterior alpha oscillations reflect attentional problems in boys with Attention Deficit Hyperactivity Disorder. Clin. Neurophysiol..

[104] Zagni E, Simoni L, Colombo D (2016). Sex and gender differences in central nervous system-related disorders. Neurosci. J..

[105] Pulvermuller F (2006). Motor cortex maps articulatory features of speech sounds. Proc. Natl. Acad. Sci..

[106] Callan D, Callan A, Jones JA (2014). Speech motor brain regions are differentially recruited during perception of native and foreign-accented phonemes for first and second language listeners. Front. Neurosci..

[107] Schomers MR, Pulvermüller F (2016). Is the sensorimotor cortex relevant for speech perception and understanding? An integrative review. Front. Hum. Neurosci..

[108] Laufs H (2003). EEG-correlated fMRI of human alpha activity. Neuroimage.

[109] Moosmann M (2003). Correlates of alpha rhythm in functional magnetic resonance imaging and near infrared spectroscopy. Neuroimage.

[110] Ritter P, Moosmann M, Villringer A (2009). Rolandic alpha and beta EEG rhythms’ strengths are inversely related to fMRI-BOLD signal in primary somatosensory and motor cortex. Hum. Brain Mapp..

[111] Goldman RI, Stern JM, Engel J, Cohen MS (2002). Simultaneous EEG and fMRI of the alpha rhythm. Neuroreport.

[112] Mullinger KJ, Mayhew SD, Bagshaw AP, Bowtell R, Francis ST (2014). Evidence that the negative BOLD response is neuronal in origin: A simultaneous EEG-BOLD-CBF study in humans. Neuroimage.

[113] Wade AR (2002). The negative BOLD signal unmasked. Neuron.

[114] Formaggio E, Storti SF, Cerini R, Fiaschi A, Manganotti P (2010). Brain oscillatory activity during motor imagery in EEG-fMRI coregistration. Magn. Reson. Imaging.

[115] Brinkman L, Stolk A, Dijkerman HC, de Lange FP, Toni I (2014). Distinct roles for alpha- and beta-band oscillations during mental simulation of goal-directed actions. J Neurosci.

